# SARS-CoV-2 and Obesity: “CoVesity”—a Pandemic Within a Pandemic

**DOI:** 10.1007/s11695-020-04919-0

**Published:** 2021-01-22

**Authors:** Kimberley Zakka, Swathikan Chidambaram, Sami Mansour, Kamal Mahawar, Paulina Salminen, Ramos Almino, Philip Schauer, James Kinross, Sanjay Purkayastha, on behalf of the PanSurg Collaborative

**Affiliations:** 1 Faculty of Medicine, American University of Beirut, Beirut, Lebanon; 2 Oxford University Clinical Academic Graduate School, Medical Sciences Office, University of Oxford, Oxford, UK; 3 Department of Surgery and Cancer, Imperial College London, St Mary’s Hospital, Academic Surgical Unit, 10th Floor QEQM, South Wharf Road, London, W2 1NY UK; 4 Sunderland Royal Hospital, Sunderland, UK; 5 Department of Surgery, Turku University, Turku, Finland; 6 Gastro-Obeso-CIMO, Sau Paulo, Brazil; 7 Pennington Biomedical Research Institute, Baton Rouge, LA USA

**Keywords:** Obesity, Coronavirus, SARS-CoV-2, COVID-19, Bariatric surgery, Obesity surgery, BMI

## Abstract

Individuals who are overweight or suffering from obesity are in a chronic state of low-grade inflammation, making them particularly susceptible to developing severe forms of respiratory failure. Studies conducted in past pandemics link obesity with worse health outcomes. This population is thus of particular concern within the context of the COVID-19 pandemic, considering the cessation of obesity management services. This systematic review highlights [1] the reciprocal link between the obesity and COVID-19 pandemics, [2] obesity as a risk factor for more severe disease in past pandemics, [3] potential mechanisms that make individual’s suffering from obesity more susceptible to severe disease and higher viral load, and [4] the need to safely resume bariatric services as recommended by expert guidelines, in order to mitigate the health outcomes of an already vulnerable population.

## Introduction

On March 11 2020, Coronavirus Disease-2019 (COVID-19) caused by the novel severe acute respiratory syndrome coronavirus (SARS-CoV-2) was declared a global pandemic by the World Health Organization (WHO) [1]. Several conditions have been associated with more severe presentations and hospital admissions, including age, male gender, obesity, hypertension, diabetes, cardiovascular disease, and chronic lung disease [2]. Recent studies have reported obesity as an independent predictive factor for worse outcomes, increased complications, and intensive care therapies [3]. While these findings enable clinicians to identify at-risk patients more promptly, it also emphasizes the neglected pre-existing obesity pandemic. The WHO estimates that obesity rates have nearly tripled worldwide since 1975 [4]. As of 2016, approximately 1.9 billion people are overweight with a body mass index (BMI) of 25–30, and over 650 million are obese with a BMI > 30 [4]. Together, this brings the global obesity rate in adults to about 45%.

Several authors have suggested a link between pandemics and subsequent increases in obesity rates [5–9]. The lockdown culture, changes in food-seeking behavior, and more sedentary home-based activities might, in effect, further worsen the obesity pandemic. The cessation of obesity management services including bariatric surgery during the pandemic could further aggravate the situation. Given suggestions that the COVID-19 pandemic may never go away, it would be particularly useful to know how individuals with obesity are faring during this viral pandemic. If they are experiencing a worse outcome, it might highlight an urgent need to double our efforts aimed at combating obesity.

To our knowledge, there is currently no systematic review in the scientific literature examining the relationship between obesity and COVID-19 pandemics. The purpose of this systematic review was to evaluate the outcomes of COVID-19 patients suffering from obesity, and the effect of the COVID-19 pandemic on the obesity pandemic itself, in accordance with the Preferred Reporting Items for Systematic Reviews and Meta-Analyses (PRISMA) guidelines [10].

## Methods

### Search Strategy and Selection of Studies

Scientific publications related to the relationship between obesity and COVID-19 infections were identified using MEDLINE, EMBASE, the Cochrane Review, and Scopus databases. Our search terms included “coronavirus” OR “COVID 19” OR “COVID-19,” “COVID-19” OR “novel coronavirus” OR “SARS-CoV-2” OR “nCoV19” OR “2019-nCoV” and a separate search using the terms “obes*” OR “overweight*” OR “over-weight*” OR “obesity” OR “BMI” OR “body mass index” OR “adipos*” OR “bariatric surgery” OR “metabolic surgery.” Both strings were combined using the AND modifier. The search included studies published up to May 16, 2020. The reference lists of eligible articles were also hand-searched for additional relevant publications.

### Selection of Studies: Inclusion and Exclusion Criteria

**Fig. 1:**
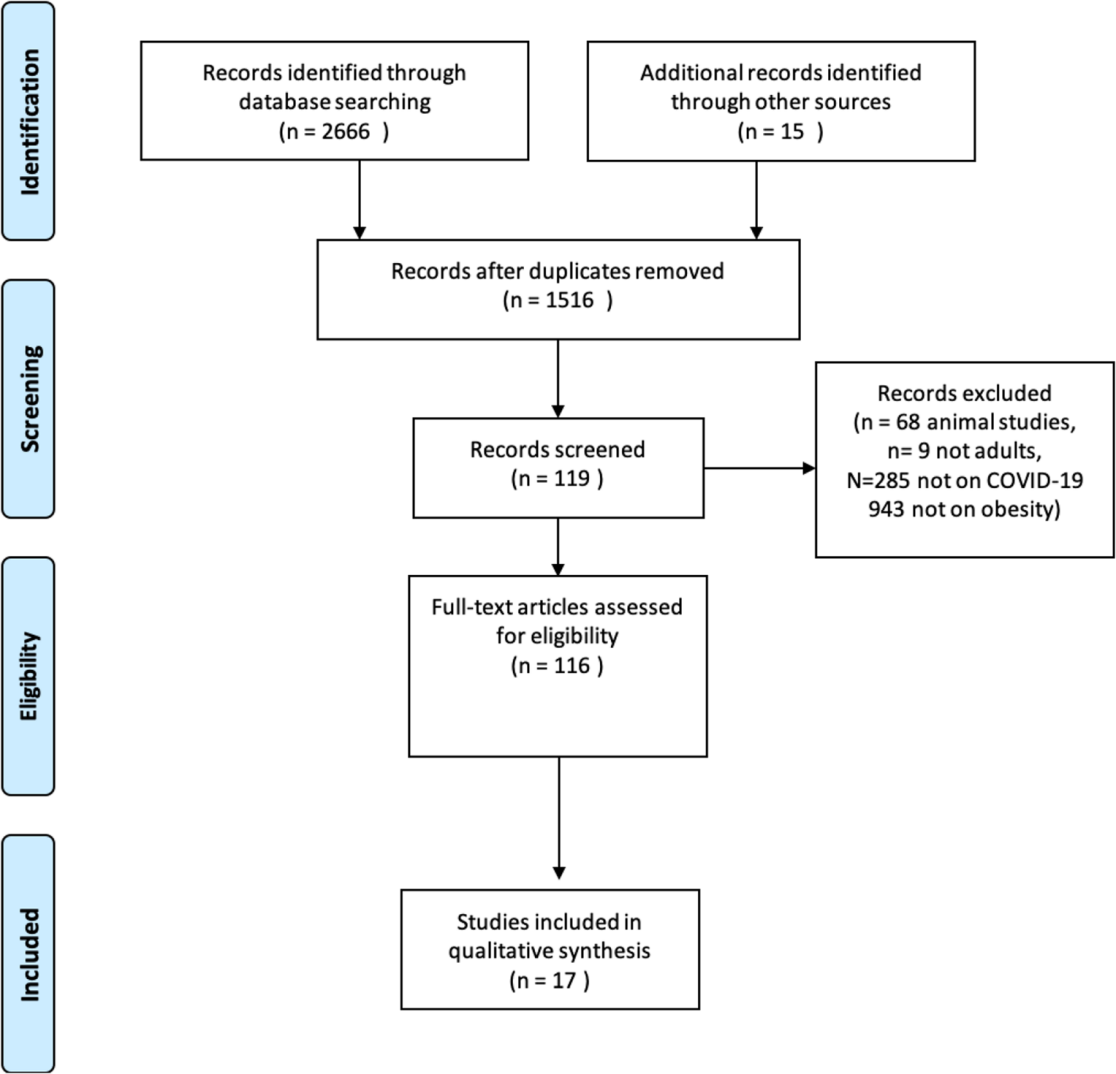
PRISMA diagram of studies

Inclusion and exclusion criteria were defined a priori. Any original study that discussed the impact of obesity on COVID-19 infections and vice versa was included. Given the limited evidence available on this topic, we included articles of all methodologies. Publications in non-English languages, non-clinical studies, and non-peer-reviewed studies (i.e., letters to the editor) were excluded. Our search yielded 2681 studies, as shown in the flow diagram (Fig. 1). After removal of duplicates, full-text articles were obtained if their abstracts were considered to be eligible. Each full-text article was assessed independently for final inclusion in this systematic review. The selection was performed by two authors (SC and KZ). In the event of conflicts of opinion, these were planned to be resolved by a third (SM).

### Data Extraction and Quality Assessment

The quality of the studies was assessed independently using the Newcastle-Ottawa score (Table 1). Disagreements regarding quality assessment score for each study were resolved by consensus. All but one study scored highly across all domains and were assessed to be of good quality. However, due to the retrospective nature of the studies included in this review, an inherent systematic bias exists. Future studies should implement more rigorous methodologies in order to more confidently draw conclusions. SC and KZ independently extracted the following data from each article using a standardized study form: (1) study information, (2) geographic location, (3) research design, (4) study objective, (5) sample size, and (6) outcomes, including admission to the intensive care unit, severity of disease, use of mechanical ventilation, and mortality.

**Table 1:** Quality assessment of studies using the Newcastle-Ottawa scale

Title	Authors	Study design	Selection	Comparability	Exposure	Quality
Obesity and COVID-19 severity in a designated hospital in Shenzhen, China	Cai et al.	Retrospective study	●●●	●	●●	Good
COVID-19 and the role of chronic inflammation in patients with obesity	Chiappetta et al.	Retrospective study	●●	●	●	Poor
Characteristics of emergency department patients with COVID-19 at a single site in Northern California: clinical observations and public health implications	Duanmu et al.	Cross-sectional study	●●	●	●●	Good
Obesity is a risk factor for greater COVID-19 severity	Goa et al.	Case-control study	●●	●	●●	Good
Association of Obesity with Disease Severity among patients with COVID-19	Kalligeros et al.	Retrospective cohort study	●●	●	●●●	Good
OpenSAFELY: factors associated with COVID-19 related hospital death in the linked electronic health records of 17 million adult NHS patients	Goldacre et al.	Retrospective cohort study	●●●●	●●	●●●	Good
High prevalence of obesity in severe acute respiratory syndrome coronavirus-2 (SARS-Cov-2) requiring invasive mechanical ventilation	Simonnet et al.	Retrospective cohort study	●●	●	●●●	Good
Clinical and chest radiography features determine patient outcomes in young and middle age adults with COVID-19	Toussie et al.	Retrospective study	●●	●●	●●	Good
Clinical characteristics of 145 patients with corona virus disease 2019 (COVID-19) in Taizhou, Zhejiang, China	Chen et al.	Retrospective study	●●	●	●●	
Bariatric surgical practice during the initial phase of COVID-19 outbreak	Aminian et al.	Retrospective cohort study	●●	●	●●●	Good
Effects of COVID-19 lockdown on lifestyle behaviors in children with obesity living in Verona, Italy: a long study	Pietrobelli et al.	Longitudinal cohort study	●●	●	●●	

## Results

### Study Characteristics (Table 2)

We found seventeen studies investigating the relationship between obesity and the COVID-19 pandemic [3, 11–25]. Of these, eleven studies evaluated the impact of obesity on COVID-19 infection, including its role as a risk factor. The remaining six studies were on how the pandemic will worsen obesity [3, 22–26]. The included studies involved a total sample size of 8,534,639 patients. All studies defined obesity as having a body mass index (BMI) ≥ 30 except for two studies performed in China on Asian populations. These investigations used a lower cut-off point for obesity of BMI ≥ 25 and BMI ≥ 28. One study used the Edmonton Obesity Staging System (EOSS) to classify its participants, noting that it is a better predictor of all-cause mortality in patients with obesity, as compared with BMI. All patients were reported to have an EOSS of 2–3 [12]. Four of the studies were policy statements from national and international organizations for bariatric surgery and hence deserved mention. The remainder was non-randomized, observational studies.

**Table 2:** Characteristics of studies including patient demographics and outcomes

Authors	Sample size	Age	Gender (male *N* (%))	BMI definition	BMI of sample population	Comorbidities	Mortality	Hospital admission	Duration of hospital stay	ICU admission	Use of ventilator
Toussie et al	338	Median age 39 (31–45)	210 (62%)	BMI > 30	130 (40%) obese or morbidly obese	Hypertension 54 (16%), asthma 46 (14%), and diabetes mellitus type II 39 (12%)	10 (7%) of admitted	145/338 admitted	29 (20%) had stay > 10 days	NA	28 (19%)
Duanmu et al	100	Median 15 (32–65)	56 (56%)	BMI > = 30	22% obese	Hypertension 19 (19%), diabetes 10%, CKD 6%, immunosuppressive meds 4%, cancer 3%, hyperlipidemia 14%, asthma 10%, COPD 1%	1%	24	Median 9 days	6%	4%
Richarson et al	5700	Median 63	60.30%	BMI > =30 obese, morbidly obese BMI > =35	1737 (41.7%) obese, 791 (19.0%) morbidly obese	Hypertension (3026;56.6%), diabetes (1808; 33.8%), CVD, cancer, chronic respiratory disease, immunosuppression, (HIV, hx organ transplant), kidney disease, liver disease	553 (21%)	All 5700	4.1 days (IQ. 2.3–6.8)	373 (14.2%)	320 (12.2%)
Kalligeros et al	103	Median 60 (52–70)	63 (61.17%)	BMI > =35 severely obese, BMI > =30 obese	49/103 (47.5%) obese	HTN (64%), diabetes (36.8%) and heart disease (24.2%)	NA	All 103	NA	41 (39.8%)	29 (28.2%)
Simonnet et al	124	Median 60 9IQR 51–70)	73%	BMI > 30 kg/m2 obese, MBMI > 35 kg/m2 severe obesity	Obesity in 47.6% and severe obesity in 28.2%	Diabetes 23%, hypertension 49%, dyslipidemia 28%	15%	All 124 in ICU	NA	124	85 (69%)
Gao et al	150	Mean age 48 years	62.70%	BMI > =25 kg/m2 obese	Obese mean BMI: 27.7; non-obese mean BMI 21.8	Diabetes (19.3%), hypertension, dyslipidemia	NA	NA	Obese (median days): 23, non-obese. 18	NA	NA
Huang et al	221	Median 45.0 (IQR. 33.5–56.0)	57%	BMI > =28 obese	Median 24.4	Sixty (27.1%) patients but at least one underlying disease including hypertension (21 [14.5%]), type 2 diabetes (21 [9.5%]), chronic lung diseases (7 [3.2%]), chronic liver diseases (6 [2.7%]), cardiovascular diseases (5 [2.3%]) cerebrovascular diseases (3 [1.4%]), and malignant tumors (3 [1.4%])	0	NA (all hospitalized)	NA	11 (5.0%)	4.10%
Goldacre et al	17 m	18 < 40 34.4%;40– < 50 16.5%;50 < 60 17.6%;60 < 70 13.8%;70– < 80:11.2%; 80 + 6.5%	49.90%	BMI 30–34.9 Obese class I. BMI 35–39.9 obese class II. BMI > =40 obese class III	13.8% BMI 30–34.9+ 5.3% BMI 35–39.9 2.7% BMI > =40	HTN (53%), respiratory diseases 4.1% (chronic heart disease 6.7%, uncontrolled DM 2.8%, controlled DM 6.0%, cancer liver. disease, neurological disease, rheumatological disease, immunosuppressive condition	< 0.01% in 18–3 years, 0.35% and 0.17% in men and women aged > = 80 years. Total 5683	NA	NA	NA	NA
Cai et al	383	N:50 (36–62); U:35.5 (28–60); OW:50 (37–61); O:48 (39–54)	N:77 (37.8); U:2 (12.5); OW:73(59.4); O:32 (78.1)	BMI < 18.5 kg/m^2^ underweight, 18.5–23.9 kg/m^2^ normal weight, 24.0–27.9 kg/m^2^ overweight, and ≥ 28 kg/m^2^ obese	53.1% normal weight (N), 4.2% underweight (U), 32.0% overweight (OW), and 10.7% obese (O)	Diabetes, hypertension, CVD, liver diseases, cancer	Did not specify	All 383 were hospitalized	Did not specify	N:16 (7.8%), U: 0; OW:14 (11.4%); O:5 (12.2%)	N:16 (7.8%); U:0; OW:14(11.4%); O: 5 (12.2%)
Chiappetta et al	33	Median 44.51 ± 11.08 (24–61)	Did not specify	Did not define	EOSS 2–3, BMI: 61.94 ± 12.3 (35–83.2)	Did not specify	NA	NA	NA	NA	NA
Chen et al	145	Mean 47.5	54.50%	Did not define	Non-severely ill, 23.20;severely ill, 24.78	HTN (15.2%), DM (9.7%), COPD, chronic liver disease, chronic kidney, peptic ulcer, solid tumor, chronic cardiac insufficiency, HIV, hyperlipidemia	NA	All 145	NA	1 (0.7%)	1 (0.7%)

## Discussion

### Obesity in Pandemics

To date, there is limited evidence elucidating the pathophysiological mechanisms linking obesity and severe COVID-19 disease. The novel coronavirus SARS-CoV-2 has 85% and 50% sequence similarity to known SARS-CoV-1 and MERS-CoV, respectively [27]. SARS-CoV-2 has been shown to gain access to the host via angiotensin-converting enzyme 2 (ACE-2) receptors which are upregulated in adipocyte and adipocyte-like cells such as pulmonary lipofibroblasts. This makes individuals with obesity and diabetes particularly vulnerable to severe lung infection and may explain the development of associated pulmonary fibrosis [28]. Moreover, low-grade systemic inflammation and end-organ damage seen in individuals with obesity are associated with the development of insulin resistance, type 2 diabetes, hypertension, atherosclerosis, dyslipidemia, and asthma—well-known comorbidities that negatively affect the outcomes of patients with COVID-19 [12]. Difficult airway management and prone positioning (critical in the treatment of ARDS) which are routinely encountered with elevated BMI further exacerbate the problem [29, 30].

This systematic review of the available evidence highlights the relationship between obesity and the COVID-19 pandemic. The included clinical characterization studies, case series, and case reports have shown that obesity is an independent, significant predictor of worse outcomes and increased complications from COVID-19 infections. Indeed, one study looked at factors associated with COVID-19 mortality by checking electronic health records of 17 million adult patients in the UK. It illustrated a positive association between patient death and degree of obesity (adjusted HR of 1.27 for BMI 30–34.9, increasing HR of 2.27 for BMI ≥ 40) [21]. Another study in a single French center analyzed 124 patients admitted in intensive care for SARS-CoV-2 and observed that obesity (BMI > 30) and morbid obesity (BMI > 35) were present in 47.6% and 28.2% of cases, respectively. Furthermore, the need for invasive mechanical ventilation (IMV) significantly was shown to increase with BMI categories, independently of age, hypertension, and diabetes—the greatest proportion of those patients (85.7%) had a BMI > 35 [20]. In the USA, a case series of 5700 patients with COVID-19 admitted to 12 hospitals in the state of New York showed that most common comorbidities exhibited were hypertension (56.6%), obesity (41.7%), and diabetes (33.8%) [13]. Another study with 103 hospitalized COVID-19-postive patients (February 17–April 5) showed that 47.5% were obese. Morbid obesity (BMI ≥ 35) was associated with ICU admission, while patients who required IMV were more likely to have had heart disease, obesity (BMI = 30–34.9), or severe obesity [19].

These findings illustrate the increased vulnerability of the population with obesity in the COVID pandemic and mirror data from past pandemics. Increased adiposity has been implicated in higher rates of complications in MERS-CoV, SARS-CoV-1, and H1N1 influenza pandemics, including development of acute respiratory distress syndrome (ARDS), acute lung injury (ALI), hospitalization, and mortality [31].

During the H1N1 swine flu of 2009, obesity was postulated to be an independent risk factor for increased morbidity and mortality in viral illnesses. In a study that spanned from April to August 2009 in California, Louie et al. found that over half of 534 adult hospitalized patients with H1N1 had a BMI > 30. Of the 92 cases who died, 61% were obese [5]. Fezeu et al. revealed that H1N1 patients with BMI > 40 were twice as likely to be admitted to the intensive care unit, with a higher mortality rate than those with a lower BMI [6].

The outbreaks of MERS and SARS in the early 2000s further established obesity as being positively associated with mortality, along with male gender, older age, hypertension, diabetes, COPD, and chronic heart diseases [9, 32, 33]. The clinical features of both of these coronaviruses, SARS-CoV-1, and MERS-CoV can range from asymptomatic or mild disease to ARDS and multi-organ failure. However, 75% of MERS cases are associated with underlying comorbidities, with a mortality rate of MERS of 60% in this subgroup, while only 10–30% of SARS patients have underlying health conditions with a mortality rate of 46% within this subgroup [34]. Given that the novel coronavirus SARS-CoV-2 has 85% and 50% sequence similarity to the SARS-CoV-1 and MERS-CoV, respectively [27], it comes as no surprise that the diseases caused by these viruses share similar clinical features [35].

### Individuals with Obesity May Be More Infectious

Several human and animal studies have demonstrated a positive correlation between infectivity and increased weight. Maier et al. demonstrated that symptomatic individuals with obesity shed influenza A particles up to 104% longer than their lean counterparts [36]. Similarly, in a study of 178 young adults, Yan et al. concluded that the concentration of viral RNA found in aerosols from collected breath correlated positively with BMI [37]. The obesity microenvironment may also be conducive to the development of more virulent viral strains. For example, obese mice models infected with influenza were observed to have a decreased type I interferon response and had increased viral replication as compared with non-obese mice [38]. There is a paucity of evidence on the infectivity of individuals with obesity in the COVID-19 pandemic, but extrapolations can be made from investigations on other viruses. While the pathophysiology of SARS-CoV2 infection has not been completely elucidated, it has been proposed that the virus gains entry into cells through ACE2-dependent mechanisms, much like SARS-CoV and human respiratory coronavirus NL63 [39]. It is spread by human-to-human transmission via droplets, aerosolized particles, and direct contact [40]. Infection has been estimated to have an incubation time varying from 2 to 14 days, with a mean of 6.4 days [40, 41]. Taking into considering prolonged viral shedding and increased viral load in expired air in persons with obesity, longer quarantine periods should be considered in individuals with increased adiposity as compared with their lean counterparts [42].

### Viral Infections May Induce Obesity

The association between obesity and viral infection is not unidirectional. Certain infections by pathogens like adenovirus 36 (Ad36) have been demonstrated to induce obesity. In a systematic review published in 2020, Kim et al. conclude that Ad36 infection increases adipogenesis (through hypertrophy and hyperplasia) in animals and is associated with human obesity. Moreover, Ad36 infection was shown to induce acute and chronic inflammation leading to angiogenesis in fatty tissues [43]. Another systematic review by Xu et al. concludes that *Helicobacter pylori* infection may be a risk factor for the development of obesity [44]. However, a causal relationship cannot be established due to the nature of the study design of the included studies.

### Indirect Impact of COVID19 on the Population Suffering from Obesity and Obesity Services

We have recognized that obesity is a risk factor for poor outcomes in COVID-19 infection [21, 45]. However, it is inevitable that the pandemic will only exacerbate the existing levels of obesity for several reasons. Firstly, the lifestyle of the average individual during a state of social isolation and global unrest will drastically change. Specifically, in the early phases of the pandemic and during lockdown measures, there will likely be a tendency towards an unhealthy diet coupled with a sedentary lifestyle. Over a longer time, this may lead to considerable weight gain for most people. Secondly, the reallocation of hospital resources from elective surgeries to managing COVID-19 patients has led to the nationwide pause of most bariatric surgery as well as most other multidisciplinary services that comprise of the tier 3 weight management programs [24]. This is not only to free up inpatient capacity and redirect healthcare workers towards managing COVID-19 patients but also to avoid intraoperative risks of viral contagion among patients and staff [22]. Hence, there is a pre-existing level of obesity that we have not managed yet. In combination, we predict a rise in obesity and its complications in the post-COVID era [46].

Globally, the majority of governments have instituted a “lockdown” approach in an effort to limit the transmission of COVID-19 cases. While this has been proven to be effective as containment measures, there are multiple negative effects of this approach. From an obesity perspective, it is highly likely that the cessation of active lifestyles will lead to an increase in weight gain among a significant proportion of the population [26]. This can be attributed to a reduction in physical exercise as well as poorer dietary lifestyle. In the early phases of the outbreak, panic buying and stockpiling depleted most stores of perishable healthy food items, including meat, fruits, and vegetables [47]. These reactions had severe repercussions on both food access and utilization [48]. Consequently, most people had to rely on unhealthy food items as sustenance. Previously, Scully et al. had shown that both lockdown and confinement would also lead to erratic dietary patterns and frequent snacking, both of which are associated with higher caloric intake and increased risk of obesity [49]. Furthermore, studies from China showed a negative impact on psychological health, which has previously been linked to unhealthy dietary patterns and poor quality of the diet [23, 50, 51]. Hence, the current lockdown is likely to increase the prevalence of obesity, and we should actively seek measures to overcome them.

The COVID-19 crisis has led to the cessation of most elective surgical procedures globally at different times. In the UK, NHS England has asked for elective procedures to be halted for 3 months starting from April 15, 2020 [52, 53]. Accordingly, early guidelines from the International Federation for the Surgery of Obesity and metabolic disorders (IFSO) recommended postponing any bariatric procedure [24]. But, delaying bariatric surgery extends the progression of metabolic complications of obesity, including type 2 diabetes, obesity hypoventilation syndrome, obesity-associated heart failure, and cancers [54–59]. This directly increases the disease burden among patients. For example, previous modeling work and the multi-cantered Swedish Obesity Study have consistently showed long-standing disease as a strong predictor of disease remission [60–62]. The impact of canceling elective bariatric surgery will be expensive. Previous economic analyses have shown that diseases that can be corrected with surgery are more cost-effective than medical management. For example, the management of type 2 diabetes with several medications is far more costly than bariatric surgery. Hence, delaying surgery for these patients will make it less cost-saving over time.

Currently, there is no large-scale data on the outcomes of bariatric surgery during the COVID-19 pandemic, and studies are ongoing to evaluate the impact of disruption of bariatric services on patient care and quality of life. However, the largest international cohort study involving surgical patients reported that complications and mortality were observed at a higher rate compared in specific patient cohorts, specifically emergency surgery, male gender, age above 70 years, and a higher ASA grade. Compared with pre-pandemic levels, morbidity and mortality is higher across all types of surgery according to this study. However, the delaying of surgery probably also leads to disease progression [63]. Hence, a realistic and safe approach will be to prioritize patients who will most benefit from surgery without exposing them to an unnecessarily high risk.

There are several solutions for the aforementioned issues. Firstly, there should be a coordinated effort from governments and food and beverage industry to ensure an adequate supply chain to prevent food insecurity. There needs to be increased public awareness about the “lockdown lifestyle” that can make them obese and provide strategies to avoid it. Already, the WHO has provided a list of exercises that can be performed at home to stay physically active [64]. Secondly, in the context of a long backlog of operations and patients with higher likelihood of complications, it is possible that there will be a shortage of staff and hospital beds to accommodate this surge. Traditionally, patients have been listed for surgery on a first-come-first-serve basis, prioritized on clinical need. Now, we must generate guidelines for prioritizing patients based on disease severity, taking into account any co-existing microvascular and macrovascular complications of obesity (indicators of organ dysfunction) [65]. For example, the Diabetes Surgery Summit (DSS) recommends that patients using insulin and patients with disease duration longer than 5 years be prioritized [25]. In the meanwhile, patients should be optimized for surgery and ensure that their weight and metabolism is controlled through lifestyle and pharmacological measures. Surgery should be expedited for patients not responding to such conservative measures. Obesity management/bariatric surgical teams must be advocates for their patients during these difficult times; otherwise, there is a significant risk of the patients’ needs being ignored due to continued public perception that obesity is still a choice and not a disease [66]. Through these measures, we may be able to mitigate the afterburn of COVID-19 on the bariatric population.

## Conclusion

COVID-19 is an unprecedented viral pandemic with high transmissibility. Obesity, a pandemic in itself, is an independent factor for having a worse outcome among COVID-19 patients. The dual pandemic—CoVesity—will have a detrimental outcome in the short, medium, and long term. We should aim at a phased and safe return of obesity/bariatric services based on expert consensus, guidelines, and recommendations from the relevant national and international bodies. There are already safe and reproducible proven interventions for the obesity pandemic that existed prior to COVID-19.
